# Impact of hyperoxia on the gut during critical illnesses

**DOI:** 10.1186/s13054-024-04848-9

**Published:** 2024-03-01

**Authors:** Ninan Dai, Juan Gu, Yanhong Luo, Yuanfa Tao, Yuehting Chou, Ying He, Han Qin, Tao Chen, Xiaoyun Fu, Miao Chen, Zhouxiong Xing

**Affiliations:** 1 Department of Critical Care Medicine, Affiliated Hospital of Zunyi Medical University, Zunyi, China; 2 Department of Pharmacy, Affiliated Hospital of Zunyi Medical University, Zunyi, China; 3 Department of Clinical Sciences, Malmö, Section for Surgery, Lund University, 214 28 Malmö, Sweden; 4 First Clinical College, Zunyi Medical University, Zunyi, China; 5 Department of Pancreatic Surgery, Renmin Hospital of Wuhan University, Wuhan, China; 6 Department of Cardiopulmonary Bypass, Wuhan Asian Heart Hospital, Wuhan, China; 7 Department of Respiratory and Critical Care Medicine, Kweichow Moutai Hospital, Guizhou Province, Zunyi, China

**Keywords:** Oxygen therapy, Hyperoxia, VA-ECMO, Gut, Gut microbiome, *Enterobacteriaceae*, Mesenteric ischemia, Anastomotic leakage, Necrotizing enterocolitis, Dual circulation

## Abstract

Molecular oxygen is typically delivered to patients via oxygen inhalation or extracorporeal membrane oxygenation (ECMO), potentially resulting in systemic hyperoxia from liberal oxygen inhalation or localized hyperoxia in the lower body from peripheral venoarterial (VA) ECMO. Consequently, this exposes the gastrointestinal tract to excessive oxygen levels. Hyperoxia can trigger organ damage due to the overproduction of reactive oxygen species and is associated with increased mortality. The gut and gut microbiome play pivotal roles in critical illnesses and even small variations in oxygen levels can have a dramatic influence on the physiology and ecology of gut microbes. Here, we reviewed the emerging preclinical evidence which highlights how excessive inhaled oxygen can provoke diffuse villous damage, barrier dysfunction in the gut, and gut dysbiosis. The hallmark of this dysbiosis includes the expansion of oxygen-tolerant pathogens (e.g., *Enterobacteriaceae*) and the depletion of beneficial oxygen-intolerant microbes (e.g., *Muribaculaceae*). Furthermore, we discussed potential impact of oxygen on the gut in various underlying critical illnesses involving inspiratory oxygen and peripheral VA-ECMO. Currently, the available findings in this area are somewhat controversial, and a consensus has not yet to be reached. It appears that targeting near-physiological oxygenation levels may offer a means to avoid hyperoxia-induced gut injury and hypoxia-induced mesenteric ischemia. However, the optimal oxygenation target may vary depending on special clinical conditions, including acute hypoxia in adults and neonates, as well as particular patients undergoing gastrointestinal surgery or VA-ECMO support. Last, we outlined the current challenges and the need for future studies in this area. Insights into this vital ongoing research can assist clinicians in optimizing oxygenation for critically ill patients.

## Background

The administration of supplemental oxygen has become one of the most commonly utilized therapies in the intensive care units (ICUs) worldwide [1, 2]. Molecular oxygen has been recognized as a “friend and foe” in treatment of critically ill patients since its discovery in the eighteenth century [3]. On the one hand, it is important for mitochondrial aerobic respiration in treating hypoxic patients; on the other hand, supplemental oxygen (e.g., oxygen inhalation and ECMO) may expose patients to supraphysiological oxygen levels, leading to arterial hyperoxia (defined as arterial oxygen partial pressure (PaO_2_) > 100 mmHg) [4, 5]. Arterial hyperoxia can provoke multiple organ injuries including the lung, retia, heart, and gut [6–9] and contribute to significantly increased mortality [10, 11].

The gastrointestinal tract is lined with a single-cell layer epithelium which has a surface area of 30 m^2^, roughly equivalent to half the size of a standard badminton court [12]. The gut houses trillions of diverse and dynamic microorganisms, termed gut microbiome (GM) [13]. Among these microorganisms, the predominant members consist of obligate anaerobes, including the classes *Clostridia* (Phylum *Firmicutes*) and *Bacteroidia* (Phylum *Bacteroidetes*), which together constitute more than 90% of the entire gut microbes [14]. It is important to note that the intestinal lumen represents an oxygen-deprived environment with an extremely low oxygen tension level, typically measuring less than 1 mmHg. This condition can be altered by the introduction of oxygen through inhalation [15]. Such an increase in oxygen availability has the potential to disrupt the stability of gut-associated microbial community and may lead to an uncontrolled expansion of oxygen-tolerant *Enterobacteriaceae* (phylum *Proteobacteria*), which is commonly recognized as a marker of dysbiosis [16, 17].

The gut and GM play key roles in the development, maintenance, and outcomes of sepsis and multiple organ dysfunction syndrome (MODS) [12, 18]. In this review, we outlined the impact of hyperoxia on the gut and GM in the context of oxygen inhalation. VA-ECMO is increasingly being used for oxygenation and circulatory support in patients with cardiogenic shock and cardiac arrest [19]. Typically, intensivists employ a peripheral cannulation strategy to facilitate rapid cannulation and provide prompt hemodynamic assistance during episodes of cardiogenic shock, with femoro-femoral VA-ECMO being the preferred approach. Additionally, we also discussed the potential repercussions of hyperoxia on the gut during peripheral VA-ECMO, as it may directly expose the intra-abdominal organs to severe hyperoxia due to dual circulation [4].

## Hyperoxia during oxygen inhalation and peripheral VA-ECMO

Oxygen inhalation is typically administered with either a conservative or liberal approach to normalize arterial hypoxia with the goal of achieving either a low or a high level of oxygenation [20]. The oxygen-ICU trial was a pivotal study that unveiled significant clinical harm associated with liberal oxygen administration, including higher mortality and increased instances of shock, liver failure, and bacteremia [20]. This evidence has led to updated clinical practice guidelines emphasizing a more conservative approach to supplemental oxygen and has spurred numerous randomized trials to define the optimal regimen for a given clinical condition [21].

**Fig. 1:**
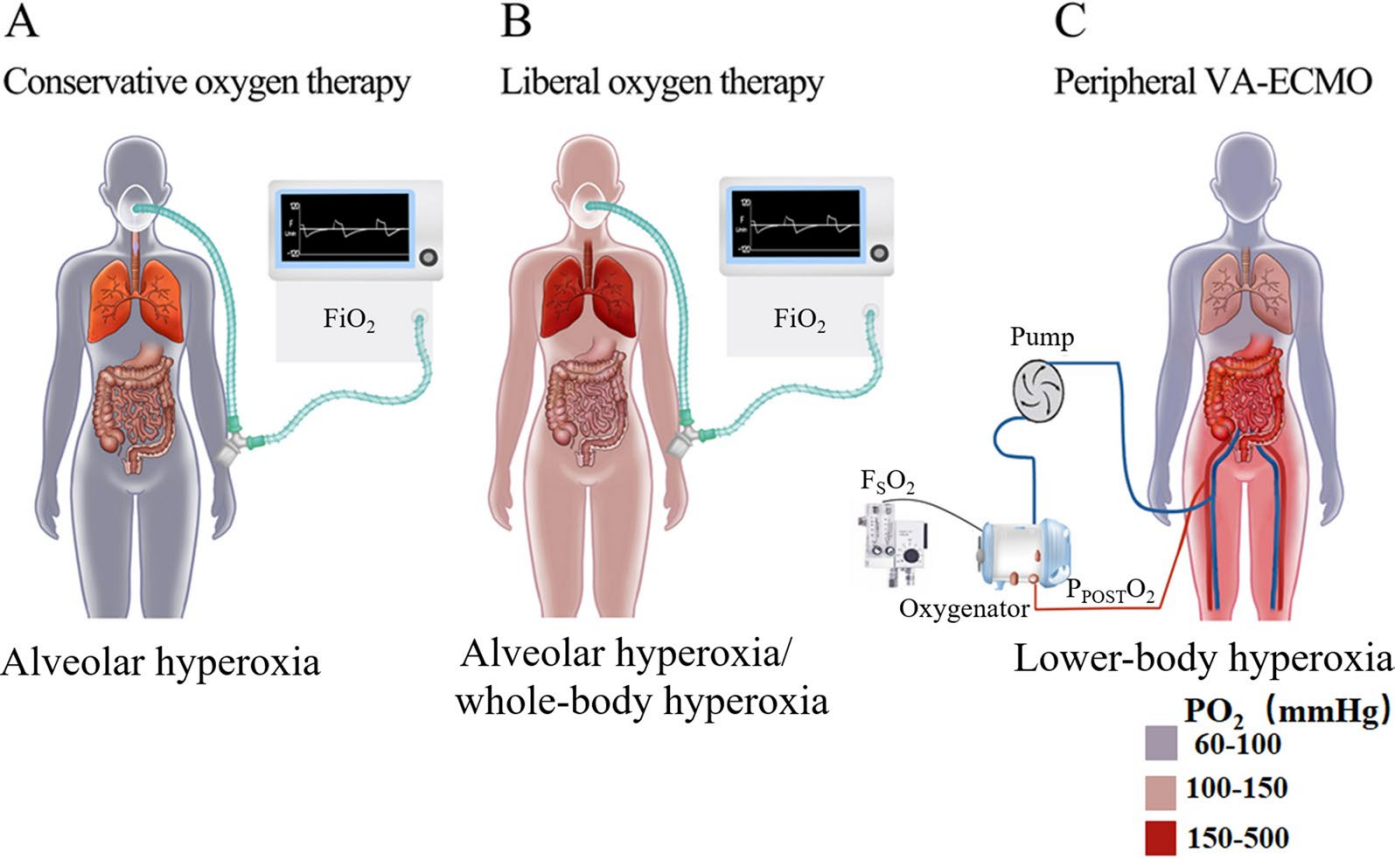
Subtypes of hyperoxia of oxygen inhalation (conservative *vs.* liberal oxygen therapy) and peripheral VA-ECMO; **A** Conservative oxygen therapy leads to mild alveolar hyperoxia and normoxemia; **B** Liberal oxygen therapy leads to moderate or severe alveolar hyperoxia and mild whole-body hyperoxia; **C** Peripheral VA-ECMO may lead to moderate or severe lower-body hyperoxia; PO_2_: partial pressure of oxygen; FiO_2_: inspired oxygen fraction; F_S_O_2_: sweep gas oxygen fraction; P_POST_O_2_: post-oxygenator oxygen partial pressure

Figure 1 illustrates the three fundamental subtypes of hyperoxia (alveolar, whole-body, and lower-body hyperoxia). These subtypes are based on the pathophysiology of traditional oxygen inhalation (conservative *vs.* liberal oxygen therapy) and peripheral VA-ECMO. Conservative oxygen inhalation involves administering oxygen at the lowest feasible inspired fraction of oxygen (FiO_2_ = 21–40%) for patients with gas exchange impairment [20]. This approach results in an elevation of oxygen tension in the bronchi, alveoli, and arterial blood, ultimately leading to mild alveolar hyperoxia and normoxemia (PaO_2_ = 70–100 mmHg) [20, 22]. Alternatively, liberal oxygen inhalation aims to maintain the FiO_2_ at least 40%, resulting in moderate to severe alveolar hyperoxia and mild whole-body hyperoxia (PaO_2_ = 100–150 mmHg) [20]. During peripheral VA-ECMO support, desaturated blood is drawn from the inferior vena cava by a centrifugal pump, saturated within an oxygenator, and reperfused in the retrograde direction back to the aorta, leading to lower-body hyperoxia [4]. The Extracorporeal Life Support Organization (ELSO) recommends targeting a post-oxygenator partial pressure of oxygen (P_POST_O_2_) around 150 mmHg during VA-ECMO [23]. However, moderate hyperoxia (PaO_2_ = 150–300 mmHg) is still observed in 30% and severe hyperoxia (PaO_2_ = 300–500 mmHg) in 20% of cardiogenic shock patients during VA-ECMO support [24, 25], and these conditions are associated with increased mortality [24, 26].

While alveolar hyperoxia is usually the primary concern during traditional oxygen inhalation [5, 27], an elevated whole-body PaO_2_ induced by liberal inspiratory oxygen may cause hyperoxia in extrapulmonary organs, including the gastrointestinal tract and GM [28, 29]. Moreover, the potential harm is directly related to hyperoxemia severity, and extreme hyperoxemia (i.e., PaO_2_ > 300 mmHg) is associated with more harm in critically ill populations [5]. During peripheral VA-ECMO, the lower body is exposed to even more severe arterial hyperoxia than during oxygen inhalation, so hyperoxic injury of the gastrointestinal tract is more likely, potentially altering the GM composition [30].

## Biological effects of intestinal hyperoxia

Molecular oxygen is vital for mitochondrial respiration and thus for cellular and tissue homeostasis. However, mitochondrial respiration also results in production of reactive oxygen species (ROS) like superoxide anion, hydroxyl radical, and hydrogen peroxide, which increase at higher oxygen concentrations [5]. These ROS have both beneficial and detrimental effects. On the one hand, ROS are involved in various enzymatic pathways, signal transduction cascades, and reparative/protective processes such as the “respiratory burst” associated with phagocytosis. However, ROS production rate surpassing the endogenous antioxidant capacity of the cell can result in oxidative damage to nucleic acids, proteins, and lipids, leading to membrane failure, mutations, and activation of programmed cell death pathways [3]. 8-hydroxy-2′-deoxyguanosine (8-OHdG) is one of the predominant forms of ROS-induced oxidative lesions, and has therefore been widely used as a biomarker for oxidative injury [31]. An animal study elucidated that hyperoxia provoked intestinal oxidative injury by increasing the level of 8-OHdG in a time- and dose-dependent manner [6].

Intestinal ischemia/reperfusion (I/R) is a common feature during sepsis and MODS [32]. Hyperoxia following ischemia and reoxygenation is due in part to the reduced affinity of hemoglobin for oxygen under elevated carbon dioxide partial pressure and/or low blood pH, termed the Bohr effect [33]. The ensuing elevation in tissue oxygen results in ROS generation and reperfusion-induced tissue damage [33]. However, there is still controversy over whether hyperoxia has beneficial of harmful effects on the intestinal I/R [34, 35]. In support of this pathomechanism, administration of superoxide dismutase, an enzyme that scavenges superoxide radicals, attenuated the increased intestinal capillary permeability induced by regional ischemia in cats [36]. Other studies reported that hyperoxia reduced intestinal vascular constriction [28] and alleviated small bowel injury after I/R [34].

## Gut injury and dysbiosis in critical illness

Over the past five decades, both clinical and experimental animal studies have highlighted the pivotal role of gut dysfunction and GM dysbiosis in critical illnesses [18]. For instance, gastrointestinal symptoms during the initial week of intensive care, especially gastrointestinal bleeding and absence of bowel sounds, were identified as independent predictors of higher 28-day mortality [37]. Similarly, intestinal fatty acid-binding protein (I-FABP), a known marker of enterocyte injury, was independently associated with shock and 28-day mortality in critically ill patients [38]. Early enteral nutrition promotes recovery in critically ill patients by preserving the homeostasis of gut barrier and GM [39]. A prevailing theory posits that gut failure increases the risk of adverse clinical outcomes by promoting sepsis and MODS [40].

Multiple studies have also reported that the GM can serve as a predictive factor for patients’ susceptibility to diseases and leveraging its potential holds promise in prevention and modulation of critical illnesses [18, 41–43]. Critical illnesses often lead to the depletion of “health-promoting” microbes and an increase in pathogenic microbes (dysbiosis) [13, 18]. Critically ill patients frequently exhibit significantly lower microbial diversity and robustness than healthy individuals. Further, opportunistic pathogens, including *Enterobacteriaceae*, *Staphylococcus*, *Enterococcus*, and *Candida albicans*, have been found to flourish in the gut of critically ill patients [13, 18, 44], while beneficial obligate anaerobes were reduced in abundance, particularly *Faecalibacterium prausnitzii* (an indicator of a healthy colonic microbiota), *Lactobacillus,* and *Bifidobacterium* [13, 18]. Probiotics and fecal microbial transplantation (FMT) have been demonstrated to improve the condition of patients with recurrent *Clostridioides difficile* infection [45, 46]. Similarly, a recent meta-analysis suggested that ingestion of probiotics or synbiotics during critical illness can reduce ventilator-associated pneumonia, healthcare-associated pneumonia, and length of stay in the ICU and hospital, although there may not be substantial effects on mortality [47].

## Animal studies

### Hyperoxia provokes gut injury and barrier dysfunction

Recent research has provided further substantiation of hyperoxia-induced gut injury in murine models through oxygen inhalation [6, 48–50]. This injury manifests as intestinal histopathological abnormalities, including mucosal atrophy (e.g., villus shortening), enterocyte death, reduced Paneth cells and goblet cells, and infiltration of polymorphonuclear leukocytes and macrophages [49, 51]. Both neonatal and adult exposure to hyperoxia disrupts the integrity of gut barrier, facilitating the translocation of lipopolysaccharides (LPS) from the gut bacteria into the bloodstream, ultimately resulting in endotoxemia [6, 51, 52]. Hyperoxia further exacerbates the cascade of gut inflammation by increasing the levels of pro-inflammatory cytokines (TNF-α, IL-1β, IFN-γ) and decreasing anti-inflammatory cytokines (IL-10, IL-17D) [49, 53]. Additionally, hyperoxia elevated the level of chemokine C-X-C motif ligand 1 in the gut epithelium, acting as a potent neutrophil chemoattractant and indicating the presence of intestinal neutrophilia [6].

**Fig. 2:**
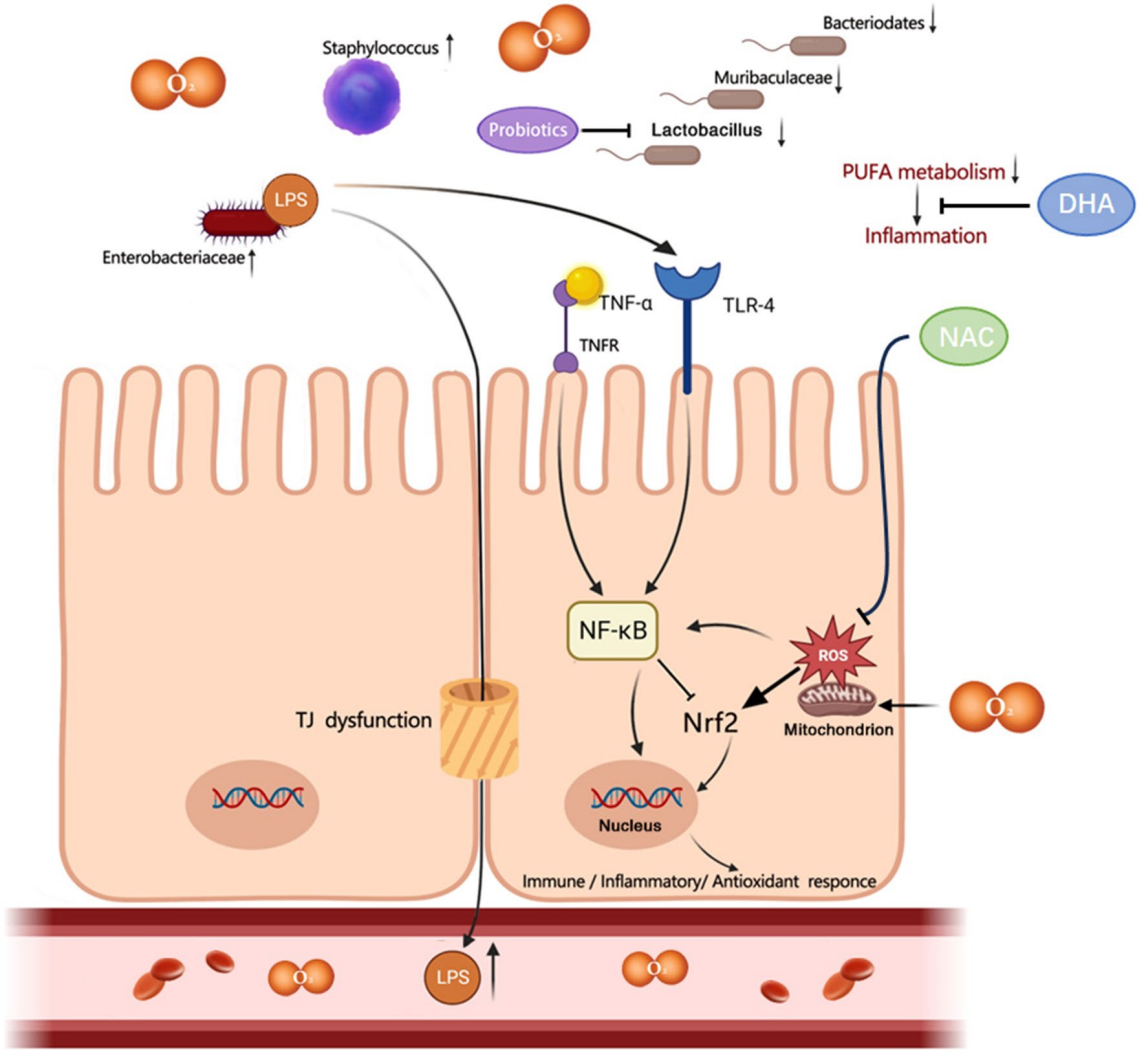
Hyperoxia provokes gut injury and dysbiosis; LPS: lipopolysaccharide; TNF-α: tumor necrosis factor-alpha; TNFR: tumor necrosis factor receptor; NF-κB: nuclear factor-κB; Nrf2: nuclear factor erythroid 2-related factor 2; TLR-4: toll-like receptor-4; NAC: N-acetylcysteine; ROS: reactive oxygen species; DHA: docosahexaenoic acid; TJ: tight junction

Hyperoxia-induced gut injury is influenced by various signaling pathways, including toll-like receptor (TLR)-4, TNF, nuclear factor-κB (NF-κB), and nuclear factor erythroid 2-related factor 2 (Nrf2) pathways [6, 49]. TLR-4 is one of the receptors involved in innate immunity activated by LPS from Gram-negative bacteria during hyperoxia [6]. Nrf2, acting as a master regulator of redox homeostasis, is upregulated by ROS and playing a crucial role in cellular protection by inducing the expression of antioxidant genes [53]. In hyperoxic conditions, NF-κB becomes activated through TLR-4 and TNF pathways, leading to the production of inflammatory cytokines, enterocyte death, and the suppression of Nrf2 [50]. Supplementation of N-acetylcysteine, a potent antioxidant, mitigates hyperoxia-induced gut injury by inhibiting ROS production [52]. In summary, oxidative stress, immunity responses, and inflammation collectively contribute to oxygen-induced gut injury (Fig. 2).

## Hyperoxia provokes gut dysbiosis and metabolic disorders

Inhalation of oxygen, leading to hyperoxia, has been extensively investigated in preclinical studies to understand its impact on the gut microbiome [29, 54–56]. Oxygen inhalation has consistently demonstrated a significant increase in *Firmicutes*/*Bacteroidetes* ratio in both neonatal and adult mice [57, 58]. Exposure to hyperoxia, both in neonatal and adult mice, has been linked to a marked expansion of oxygen-tolerant microbes, particularly pathogenic *Proteobacteria*, *Enterobacteriaceae,* and *Staphylococcus* [6, 29, 56]. Concurrently, it has led to a decrease in the relative abundance of oxygen-intolerant microbes like *Bacteroidetes* and *Muribaculaceae*, as well as *Lactobacillus* in mice [6, 55, 57]. Notably, even a brief exposure to hyperoxia (72 h) in adult mice resulted in significant alterations in gut microbiome, marked by the depletion of *Ruminococcaceae* [29]. *Muribaculaceae*, *Lactobacillus*, and *Ruminococcaceae* are beneficial microbes which can produce short chain fatty acids (SCFAs) with anti-inflammatory properties [59–61]. Hyperoxia may reprogram the metabolic pathways in the gut producing the abnormal metabolites [9]. Our recent metabolomics analysis revealed that oxygen inhalation suppressed the metabolism of fecal polyunsaturated fatty acids (PUFAs) in mice, resulting in reduced levels of linoleic acids and α-linolenic acids, along with their secondary metabolites [57]. Supplemental docosahexaenoic acid (DHA), a beneficial PUFA, may limit inflammation and apoptosis involved in hyperoxia-induced intestinal injury in neonatal mice [62]. These findings collectively suggest the presence of gut dysbiosis and metabolic disorders induced by hyperoxia [9, 49] (Fig. 2).

## Cross talk between hyperoxia-induced gut dysbiosis and distant organ injuries

**Fig. 3:**
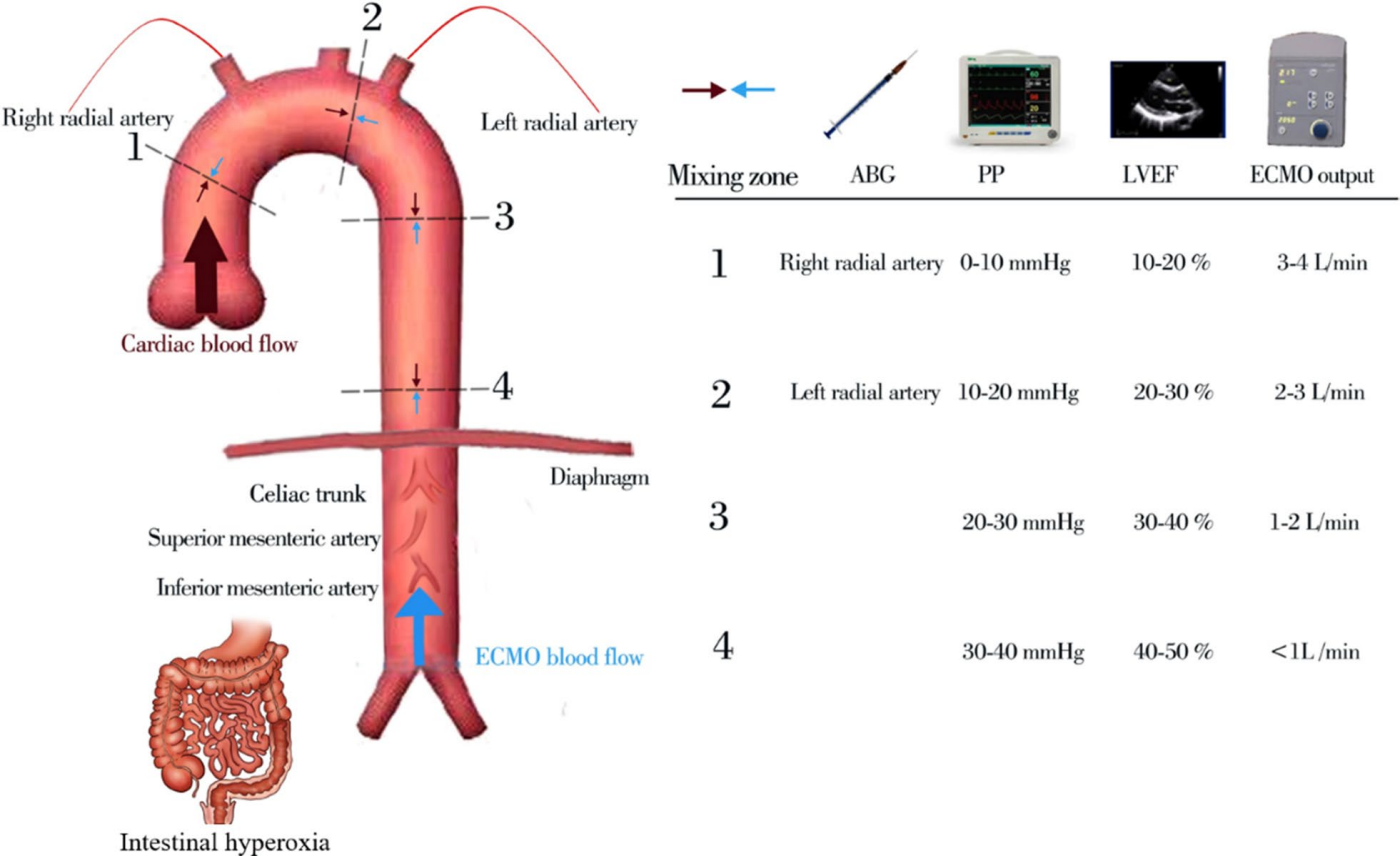
Dual circulation, mixing zone, and intestinal hyperoxia in peripheral VA-ECMO; PP: pulse pressure; ABG: arterial blood gas; LVEF: left ventricular ejection fraction; Mixing zone 1: ascending aorta; 2: aortic arch; 3: descending aorta; 4: thoracic/abdominal aorta

The GM contributes to the development and function of remote organs, including the lungs through the “gut–lung axis” and brain through the “gut–brain axis” [63]. Hyperoxia can disrupt GM homeostasis and proper signaling through these axes, contributing to gut, lung, and brain dysfunction [6, 54, 64]. For instance, hyperoxia was reported to promote bacterial translocation from the gut to the lungs in neonatal mice [65]. Oxygen inhalation also decreased the relative abundance of intestinal *Lactobacillus*, while administration of *Lactobacillus* attenuated hyperoxia-induced lung injury in murine models [55, 66]. Alternatively, oral administration of antimicrobial peptides restored gut dysbiosis and alleviated lung injury in mice exposed to hyperoxia [56]. Variations in gut bacterial communities correlated with variations in lung inflammation among hyperoxia-exposed mice [29]. Adopting a high-fiber diet or supplementation of the gut microbe metabolite acetate (SCFA) increased the abundance of *Bacteroides*, thereby mitigating gut dysbiosis and attenuating acute lung injury in hyperoxia-exposed mice [67]. In addition, it was reported that the TLR-4 pathway may contribute to the deleterious effects of hyperoxia-induced gut dysbiosis on lung development [64]. Hyperoxia exposure during the first week of life and ensuing gut dysbiosis were also implicated in brain dysfunction at adolescence in mice [54]. Collectively, these preclinical studies suggest that intestinal inflammation, dysbiosis, and metabolic disturbances contribute to oxygen-induced lung injury and brain dysfunction through gut–lung and gut–brain axes [9] (Fig. 3).

## Clinical studies

A literature search was conducted in the PubMed database using the following keywords: “oxygen therapy,” “hyperoxia,” “hyperoxemia,” “anastomotic leakage,” “necrotizing enterocolitis,” and “ECMO.” Table 1 presents the key clinical studies that investigated the impact of hyperoxia on the intestinal morbidity. Notably, there is a dearth of human studies focusing on the effects of hyperoxia during VA-ECMO on the gut. Consequently, this discussion delves into the correlation between intestinal hyperoxia and dual circulation during peripheral VA-ECMO.

**Table 1:** Impact of hyperoxia on gut-associated morbidity in clinical studies

Study name (sample size)	Settings	Lower versus higher oxygenation targets	Primary outcomes	Gut-associated morbidity
LOCO2 [69]	Acute respiratory distress syndrome in adults	SPO_2_ = 88- 92% *vs.* SPO_2_ ≥ 96%	The conservative oxygenation strategy did not increase survival at 28 days	Five mesenteric ischemic events occurred in the conservative oxygen group
(*n* = 205)				
HOT-ICU [70]	Acute hypoxemic respiratory failure in adults	PaO_2_ = 60 mmHg *vs.* PaO_2_ = 90 mmHg	No significant difference in 90-day mortality between the two groups	No significant difference in intestinal ischemia between the two groups
(*n* = 2928)				
BOOST NZ [77]	Preterm infants	SpO_2_ = 85%-89% *vs.* SpO_2_ = 91%-95%	No significant difference in mortality between the two groups	No significant difference in NEC between the two groups
(*n* = 340)				
BOOST II AUS [78]	Preterm infants	SpO_2_ = 85%-89% *vs.* SpO_2_ = 91%-95%	No significant difference in mortality between the two groups	No significant difference in NEC between the two groups
(*n* = 1135)				
BOOST II UK [79]	Preterm infants	SpO_2_ = 85%-89% *vs.* SpO_2_ = 91%-95%	Lower oxygenation target was associated with increased mortality	Lower oxygenation target increased the incidence of NEC
(*n* = 2448)				
COT [80]	Preterm infants	SpO_2_ = 85%-89% *vs.* SpO_2_ = 91%-95%	No significant difference in mortality between the two groups	No significant difference in NEC between the two groups
(*n* = 1201)				
SUPPORT [76]	Preterm infants	SpO_2_ = 85%-89% *vs.* SpO_2_ = 91%-95%	No significant difference in mortality between the two groups	No significant difference in NEC between the two groups
(*n* = 1316)				
Meta-analysis by Askie et al. [75]	Preterm infants	SpO_2_ = 85%-89% *vs.* SpO_2_ = 91%-95%	Lower oxygenation target was associated with increased mortality	Lower oxygenation target increased the incidence of NEC
(*n* = 4965)				
Meta-analysis by Kuh et al. [94]	Gastrointestinal surgery in adults	FiO_2_ = 30% *vs.* FiO_2_ = 80%	High FiO_2_ reduced the incidence of SSI	High FiO_2_ decreased the anastomotic leakage incidence
(*n* = 15,877)				
PROXI [95]	Acute or elective laparotomy in adults	FiO_2_ = 30% *vs.* FiO_2_ = 80%	No significant difference in SSI incidence between the two groups	No significant difference in anastomotic leakage between the two groups
(*n* = 1400)				

NEC: necrotizing enterocolitis; SSI: surgical site infection

## Lower *vs.* higher oxygenation targets and intestinal morbidity in adults

The recent LOCO2 trial, a multicenter randomized trial, studied liberal oxygen therapy (higher oxygenation targets) or conservative oxygen therapy (lower oxygenation targets) in patients with acute respiratory distress syndrome (ARDS) [68]. The trial was prematurely halted due to increased 90-day mortality and concerns about five episodes of mesenteric ischemia, all occurring in the conservative oxygen group [1]. However, subsequent analysis in the HOT-ICU trail revealed no significant differences in 90-day mortality or incidence of new episodes of intestinal ischemia between the lower and higher oxygenation target groups in the broader ICU population [69]. Further, the HOT-ICU trial included a sample approximately 15-fold larger than the LOCO2 trial, suggesting that the unequal distribution of intestinal ischemia between groups in the LOCO2 trial occurred by chance [70]. In addition, the increased incidence of mesenteric ischemia in the LOCO2 trial may be attributed to the lower oxygenation target (a peripheral oxygen saturation, SpO_2_ = 88%) in the conservative arm compared to that in the HOT-ICU trial (SpO_2_ = 90%) [1].

While the current studies on liberal oxygen inhalation do not report gut-associated adverse events [71], the possibility of subclinical enterocyte injury, such as elevated serum level of I-FABP, cannot be entirely ruled out. The sensitivity of small bowel to reduced oxygen delivery is well established owning to its unique blood supply characteristics [72]. Therefore, special attention should be given to the potential occurrence of intestinal ischemia, particularly in patients more susceptible to hypoxia, such as those with ARDS, when prescribing conservative oxygen therapy [1].

## Lower* vs.* higher oxygenation targets and intestinal morbidity in infants

Necrotizing enterocolitis (NEC) is among the most common and devastating diseases in infants [73]. The Neonatal Oxygen Prospective Meta-analysis Collaboration, established in 2003 [74], was a joint effort involving investigators from five distinct randomized clinical trials: SUPPORT trial [75], the three BOOST II trials conducted in the UK, Australia, and New Zealand [76–78], and the COT trial [79]. These five studies, with a total sample size of almost 5000 infants, were designed to compare the effects of a lower oxygen saturation target (SpO_2_ = 85–89%) versus a higher target (SpO_2_ = 91–95%) on death or major disability as the primary outcome. According to the results, a lower oxygen saturation target significantly increased the mortality rate and the ratio of NEC patients requiring surgery [74]. NEC is more likely to occur in the colon, which has a higher ischemic tendency because of its dependence on the most distal branches of the mesenteric vascular supply [80]. However, the clinically appropriate oxygen saturation range in the intestines for preterm infants is unknown and may vary with gestational and postnatal age [81].

## Perioperative hyperoxia and gastrointestinal surgery

Resection and anastomosis of the digestive tract is a commonly carried out procedure during gastrointestinal surgery [82]. Anastomotic leakage is a major postoperative complication which can result in local collections and sepsis and increase the length of hospital stay and mortality in patients [83, 84]. Tissue oxygen tension is often low in wounds and colorectal anastomoses that may reduce tissue healing and lead to a high risk of surgical site infection (SSI) and anastomotic leakage [85–87]. In 2016, the World Health Organization (WHO) recommended supplementing 80% oxygen during anesthesia and, if feasible, for 2 to 6 h after surgery to reduce SSI risk [88]. However, these WHO guidelines have met criticism due to the weakness of the underlying analyses [89]. Further, two updated meta-analyses and further clinical trials with large sample sizes did not detect any benefit of perioperative hyperoxia, but instead observed an increased frequency of pulmonary complications [90–92]. A recent meta-analysis did find reduced SSI and anastomotic leakage incidences in the pooled high FiO_2_ arm, but this finding should be interpreted with caution due to low quality of evidence presented in the individual studies [93]. Moreover, the double-blinded randomized PROXI trial of 1400 patients found that administration of 80% oxygen did not result in a significant change in the risks of SSI and anastomotic leakage after abdominal surgery compared to 30% oxygen [94].

Given this emerging evidence for the detrimental effects of hyperoxia on the gut [49], even with exposure for a brief 24-h period [6], perioperative hyperoxia may not be suitable during gastrointestinal surgery. In fact, the World Federation of Societies of Anesthesiologists recommends conservative perioperative oxygen inhalation (FiO_2_ = 30–40%) for intubated patients during general anesthesia and a normal SpO_2_ above 93% postoperatively [89, 95].

## Dual circulation during peripheral VA-ECMO and intestinal hyperoxia

**Fig. 4:**
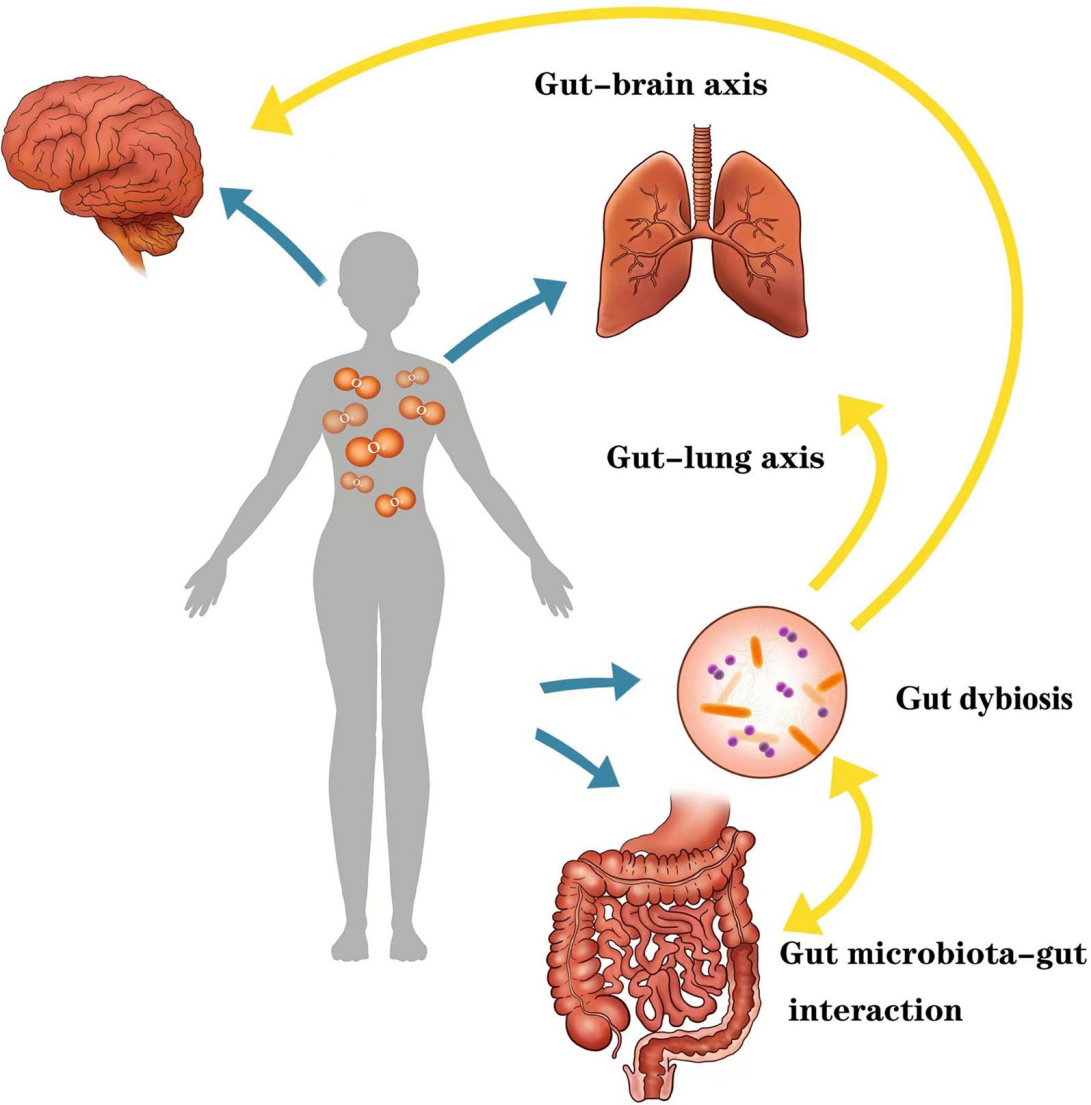
Cross talk between hyperoxia-induced gut dysbiosis and distant organ injuries

Peripheral VA-ECMO creates a dual-circulation hemodynamic pattern with competitive blood flows from both the heart and VA-ECMO contributing to circulation and oxygenation [4]. The mixing zone is defined as the point where these two flows intersect and the specific location of this mixing zone is contingent on the ratio of native cardiac output (NCO) to ECMO blood flow [96]. As NCO increases while ECMO support decreases, the mixing zone shifts distally along the aorta [96]. Arterial blood gas (ABG), pulse pressure (PP), and point-of-care ultrasound are valuable parameters for NCO assessment [96–98]. Using these methods, the mixing zone can be approximately localized to the ascending aorta if the NCO/ECMO flow ratio is 1:4, to the aortic arch if 1:3, the descending aorta if 1:2, and thoracic aorta/abdominal aorta if 1:1 [99]. The mesenteric circulation is primarily supplied by the celiac trunk and superior and inferior mesenteric arteries [100]. Celiac trunk mixing (50%/50% from the NCO/ECMO) occurs when the NCO/ECMO flow ratio is 1:1 [99]. These findings demonstrate that patients on VA-ECMO support are frequently exposed to prolonged hyperoxia of the gastrointestinal tract (Fig. 4).

## Current challenges and future studies

Most preclinical studies have reported marked hyperoxia-induced gut injury and dysbiosis after exposing healthy animals to inhaled oxygen with a very high FiO_2_ (e.g., > 80%) [29, 48, 56]. However, this condition rarely occurs in clinical practice when oxygen inhalation is prescribed for hypoxic patients [21]. Therefore, it may be more fruitful to assess the impact of hyperoxia on the gastrointestinal tract in animal disease models (e.g., mice with acute lung injury) and other clinical conditions such as ARDS.

In contrast to inhaled oxygen, VA-ECMO directly exposes the human gastrointestinal tract to hyperoxia, potentially inducing gut injury and dysbiosis [4, 30]. Further animal model and clinical studies are needed to assess the frequency, severity, and risk factors for this condition. Most recent studies have detected arterial hyperoxia by measuring ABGs in samples extracted from the right arm as per the institutional ECMO protocol, primarily due to concerns regarding Harlequin syndrome [25, 101, 102]. The syndrome describes when NCO recovery coexists with respiratory failure, an antegrade ejection by the left ventricle of relatively deoxygenated blood hinders the retrograde oxygenated blood flow of the VA-ECMO in the aortic arch resulting in upper-body hypoxia [103]. As a result of dual circulation, however, the PaO_2_ measured at the right arm may underestimate the oxygen burden in the lower body [30]. We therefore suggest that future studies adopt P_POST_O_2_ act as a metric for hyperoxia assessment and concomitantly measure markers of gut injury (e.g., I-FABP), dysbiosis (e.g., *Enterobacteriaceae*) and metabolic disorders (e.g., SCFAs) to assess the potential negative impact of intestinal hyperoxia.

The impact of hyperoxia on the gut and GM appears to vary according to the clinical situation. While hyperbaric hyperoxia may be associated with improved anastomotic healing and a decreased risk of inflammatory bowel disease flares [86, 104], excess normobaric hyperoxia has harmful effects on the gut [6]. Maturation of oxygenation is a dynamic process in the developing intestines of infants and differs from adults [81]. Future studies should thus address how a particular oxygenation target can be set and achieved in specific age and illness groups, and how the particular target influences the gut and GM [1].

Finally, emerging novel technologies may help clinicians improve the regulation of intestinal oxygenation during oxygen therapy. Abdominal near-infrared spectroscopy can be used to measure intestinal oxygenation changes and to diagnose NEC in preterm infants [105, 106]. Several devices have also been developed to continuously monitor the P_POST_O_2_ during VA-ECMO support [4]. The reliability of these devices for reducing lower-body hyperoxia and minimizing hyperoxic injury to the intestines must be confirmed.

## Conclusions

Recent animal studies highlight the risk of gut injury and dysbiosis induced by excessive oxygen inhalation. While the effects of hyperoxia on gut integrity and GM composition may vary across clinical settings and patient conditions, lower oxygenation targets may increase the risk of mesenteric ischemia and NEC in adults (SpO_2_ = 88–92%) and infants (SpO_2_ = 85–89%), respectively. Further, perioperative hyperoxia (FiO_2_ = 80%) may not reduce the risk for SSI and anastomotic leaks during gastrointestinal surgery, so a lower oxygenation target (FiO_2_ = 30–40%) may be more rational. Extreme intestinal hyperoxia (PaO_2_ ≥ 300 mmHg) during VA-ECMO is likely to contribute to gut injury and dysbiosis, and further studies are needed. It appears that targeting oxygenation within a normal range may help to avoid both hyperoxia-induced gut injury and hypoxia-induced mesenteric ischemia among most ICU patients. In addition, we suggest that PaO_2_ should be carefully titrated for specific clinical situations and contexts.

## Data Availability

Not applicable.

## Abbreviations

VA-ECMO: Venoarterial-extracorporeal membrane oxygenation
ROS: Radical oxygen species
GM: Gut microbiome
ICUs: Intensive care units
ELSO: Extracorporeal Life Support Organization
P_POST_O_2_: Post-oxygenator oxygen partial pressure
MODS: Multiple organ dysfunction syndrome
FiO_2_: Inspired oxygen fraction
PO_2_: Oxygen partial pressure
PaO_2_: Arterial oxygen partial pressure
SpO_2_: Peripheral oxygen saturation
NCO: Native cardiac output
ABG: Arterial blood gas
PP: Pulse pressure
I-FABP: Intestinal-fatty acid-binding protein
ARDS: Acute respiratory distress syndrome
NEC: Necrotizing enterocolitis
SSI: Surgical site infection
WHO: World health organization
I/R: Intestinal ischemia/reperfusion
SCFAs: Short chain fatty acids
PUFAs: Polyunsaturated fatty acids
8-OHdG: 8-Hydroxy-2′-deoxyguanosine
